# Construction of an Immunogenomic Risk Score for Prognostication in Colon Cancer

**DOI:** 10.3389/fgene.2020.00499

**Published:** 2020-05-21

**Authors:** Han Zhang, Chuan Qin, Hua Gan, Xiong Guo, Li Zhang

**Affiliations:** ^1^First Clinical Medical College, Chongqing Medical University, Chongqing, China; ^2^Department of Digestive Oncology, Three Gorges Hospital, Chongqing University, Chongqing, China; ^3^Department of Gastrointestinal Surgery, Three Gorges Hospital, Chongqing University, Chongqing, China

**Keywords:** immune-related genes, immune system, colon cancer, prognostication, biomarkers

## Abstract

Immune-related genes (IRGs) play regulatory roles in the immune system and are involved in the initiation and progression of colon cancer. This study aimed to develop an immunogenomic risk score for predicting survival outcomes among colon cancer patients. We analyzed the expressions of IRGs in colon specimens and discovered 484 differentially expressed IRGs when we compared specimens from colon cancer and adjacent normal tissue. Univariate Cox regression analyses were performed to identify 26 IRGs that were associated with survival. A Cox proportional hazards model with a lasso penalty identified five optimal IRGs for constructing the immunogenomic risk score (*CD1B, XCL1, PLCG2, NGF*, and *OXTR*). The risk score had good performance in predicting overall survival among patients with colon cancer and was correlated with the amount of tumor-infiltrating immune cells. Our findings suggest that the immunogenomic risk score may be useful for prognostication in colon cancer cases. Furthermore, the five IRGs included in the risk score might be useful targets for investigating the initiation of colon cancer and designing personalized treatments.

## Introduction

Colorectal cancer (CRC) is the third most common malignancy and the second leading cause of cancer-related death, with an estimated 1,800,000 new diagnoses and 881,000 deaths in 2018 (Bray et al., 2018). Despite advances in diagnosis and treatment, the 5-year survival rate remains approximately 57% (Holleczek et al., 2015), which highlights the need for strategies to provide better outcomes. The tumor-node-metastasis (TNM) system is an essential prognostic tool for guiding treatment selection (Hu et al., 2011; Kim et al., 2011; Park et al., 2012), although some patients with the same disease stage experience different survival outcomes, which is related to molecular heterogeneity. Therefore, it is critical to identify biomarkers that can help predict the risks of recurrence and death, which can facilitate early interventions and improve outcomes among patients with colon cancer.

There is increasing evidence that dysregulation of the immune system is involved in the initiation and progression of cancer (Fridman et al., 2012; Janssen et al., 2017). For example, cancer cells can escape immune system recognition and elimination by upregulating and downregulating immune-related genes (IRGs), which help promote tumor growth (de Vries et al., 2016). Many reports have also confirmed that IRGs are attractive targets for regulating cancer progression (Weß and Schnieders, 2017; Cabrero-de Las Heras and Martínez-Balibrea, 2018). Therefore, IRG-based features may be useful for prognostication in CRC cases.

Several studies have evaluated the relationships between IRG-based genetic signatures and the prognosis of CRC (Ge et al., 2019; Wu et al., 2019). However, there are significant differences between colon and rectal cancers in terms of their embryological origin, anatomy, and functional implications (Tamas et al., 2015; Riihimäki et al., 2016; Paschke et al., 2018). Tumor-based heterogeneity is also apparent between individuals and between tumor sites (Imperial et al., 2018), and we suspect that IRG expression profiles also vary between colon and rectal cancers. However, we are not aware of any studies that have evaluated the characteristics and regulatory mechanisms of IRGs in colon cancer. Therefore, this study aimed to evaluate IRGs in colon cancer and to develop an immunogenomic risk score to predict the prognosis of patients with colon cancer.

## Materials and Methods

### Data Retrieval

Transcriptome expression profiles for colon cancer samples were downloaded from The Cancer Genome Atlas data portal (TCGA)^1^ (Liu et al., 2018). Clinical data for the corresponding patients were also retrieved from the database, which included sex, age, tumor stage, and survival information. Patients without survival data or with <30 days of data were excluded because they might have died because of lethal complications (e.g., digestive tract infection or hemorrhage), rather than colon cancer. The expression of protein-coding genes were annotated in the TCGA data portal based on fragments per kilobase of transcript per million mapped reads (FPKM).

Data regarding 2,498 IRGs were downloaded from the Immunology Database and Analysis Portal database^2^ (Bhattacharya et al., 2018). Data regarding cancer-associated transcription factors (TFs) were also obtained from the Cistrome project^3^ (Mei et al., 2017). Immune infiltrate data were collected from the Tumor Immune Estimation Resource^4^ (Li et al., 2017), which contains information regarding the relative proportions of six types of tumor-infiltrating immune cells (B-cells, CD4^+^ T-cells, CD8^+^ T-cells, neutrophils, macrophages, and dendritic cells).

### Differential Gene Analysis

Genes and TFs that were differentially expressed between colon cancer specimens and adjacent normal colon specimens were identified using the “limma” package for R software, with a |log2 fold-change [logFC]| of >1 and an adjusted false-discovery rate (FDR) of <0.05. Differentially expressed IRGs were identified among the differentially expressed genes, with expression patterns of significant differentially expressed genes and IRGs visualized using heatmaps and volcano plots. These elements were created using the “pheatmap” package for R software.

### Functional Enrichment Analysis of the Differentially Expressed IRGs

Biological processes, molecular functions, and cellular components that were potentially associated with the differentially expressed IRGs were evaluated using Gene Ontology data (Ashburner et al., 2000) from the Database for Annotation, Visualization and Integrated Discovery (version 6.8)^5^. These data were analyzed using the “goplot” package for R software. Enrichment analysis was performed using the Kyoto Encyclopedia of Genes and Genomes (Kanehisa et al., 2017) and data from the KOBAS database (version 3.0)^6^ (Xie et al., 2011). These data were analyzed using the “clusterProfiler” package for R software.

### Construction of a TF Regulatory Network

Regulatory mechanisms were evaluated by screening TFs that were differentially expressed between colon cancer and normal colon tissues (*P*-values of <0.05). We identified clinically relevant TFs based on a correlation assessment (correlation coefficient >0.4), and constructed a regulatory network of the relevant IRGs and potential TFs using Cytoscape software (version 3.7.2) (Su et al., 2014).

### Development and Validation of the Risk Score

The patients were randomly assigned to a training dataset and a testing dataset. The training dataset was used to develop the risk score. Survival-associated IRGs were identified using univariate Cox analyses and the “survival” package for R software (*P*-value <0.01). Next, a Cox proportional hazards model with a lasso penalty was used to identify the genetic model with the best prognostic value, which was performed using the “glmnet” and “survival” packages for R software. The risk score was then created based on the gene model, using the Gene Expression Profiling Interactive Analysis website^7^ (Tang et al., 2017) to analyze the expression of each IRG that was included in the risk score. The risk score was calculated as:

$$R\,i\,s\,k\,s\,c\,o\,r\,e=\sum_{i=1}^{N}\left(\mathrm{Expi}\times \mathrm{Coef}\right)$$

with N representing the number of signature genes, Expi representing the gene expression levels, and Coef representing the estimated regression coefficient value from the Cox proportional hazards analysis.

The risk score’s predictive value was evaluated using receiver operating characteristic (ROC) curve analysis in the training and testing datasets. For that analysis, an area under the ROC curve (AUC) of >0.75 was judged to have excellent predictive value. The predictive value was also evaluated by grouping patients into high-risk and low-risk groups (based on the median risk score), with univariate and multivariate analyses of survival then performed for the risk score and clinical factors. We also evaluated the relationships of the risk score with clinical data and tumor-infiltrating immune cells using a correlation assessment (significant at a *P*-value of <0.01).

### Statistical Analysis

The data were analyzed using R software (version 3.6.1)^8^. Differentially expressed genes were identified using the Wilcox test. Survival analyses were performed using the Kaplan-Meier method and the log-rank test.

## Results

### Identification of Differentially Expressed IRGs

**FIGURE 1:**
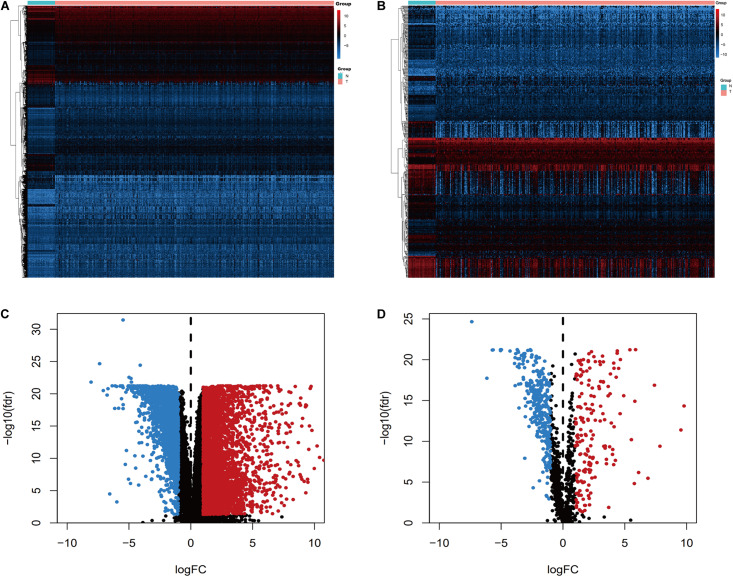
Expression of genes and IRGs. Heatmap **(A)** and volcano plot **(C)** showing the differentially expressed genes between colon cancer and normal colon specimens. Heatmap **(B)** and volcano **(D)** showing the differentially expressed IRGs. Red dots represent upregulated and blue dots represent downregulated differentially expressed genes, and black dots represent no difference.

**FIGURE 2:**
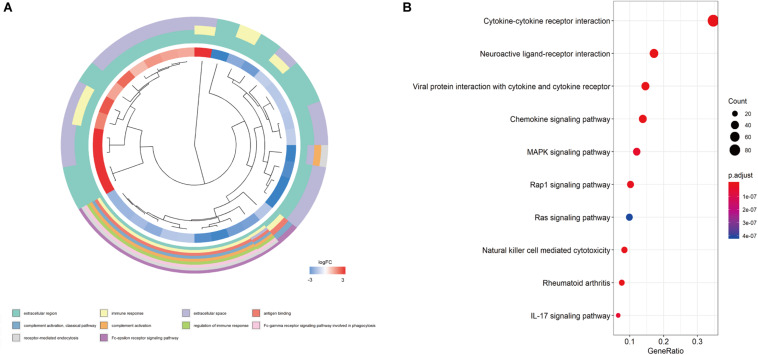
Functional enrichment of differentially expressed IRGs. **(A)** Gene Ontology analyses; the red region represents upregulated IRGs, and the blue region represents downregulated IRGs. **(B)** The top 10 most significant Kyoto Encyclopedia of Genes and Genomes pathways.

We screened the expression levels of 60,483 genes in colon cancer specimens (*n* = 398) and normal colon tissues (*n* = 39). This screening identified 6,501 differentially expressed genes, including 4,478 genes that were upregulated and 2,023 genes that were downregulated, relative to the levels in normal colon tissues (log2 fold-change [logFC] of >1.0, FDR of <0.05) (Figures 1A,C). We searched these differentially expressed genes and identified 484 differentially expressed IRGs, including 173 upregulated IRGs and 311 downregulated IRGs (logFC of >1.0, FDR of <0.05) (Figures 1B,D). All of the IRGs are protein-coding genes. The Gene Ontology analyses of differentially expressed IRGs revealed that “immune response” was the most common biological process, “extracellular region” was the most common cellular component, and “antigen-binding” was the most common molecular function (Figure 2A). The Kyoto Encyclopedia of Genes and Genomes pathway analyses revealed that most of the IRGs played roles in cytokine-cytokine receptor interactions (Figure 2B).

### Identification of Survival-Associated IRGs

**FIGURE 3:**
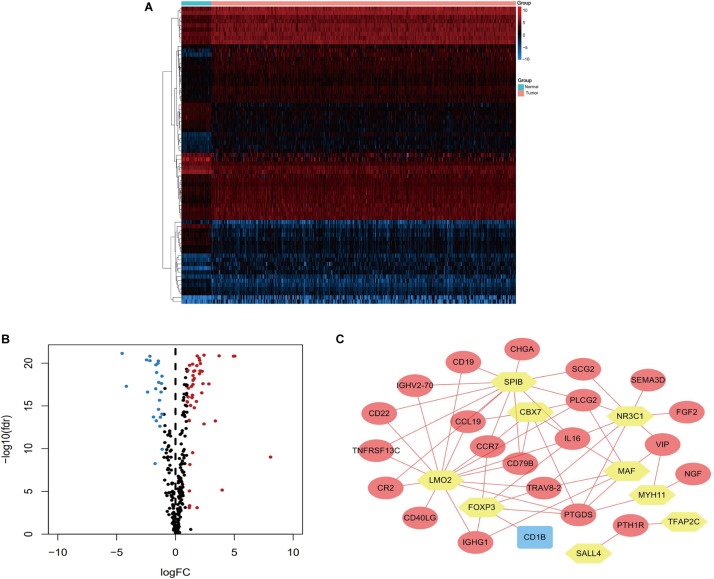
Heatmap **(A)** and volcano plot **(B)** showing the differentially expressed TFs between colon cancer and normal colon specimens. Red dots represent upregulated and blue dots represent downregulated differentially expressed TFs, and black dots represent no difference. **(C)** The intersection of differentially expressed TFs and survival-associated IRGs. Yellow hexagons represent TFs. Rectangles and ovals represent IRGs.

Univariate Cox regression analyses were performed for the differentially expressed IRGs, which revealed that 26 IRGs were significantly associated with overall survival among colon cancer patients (*P* < 0.01) (Table 1). Their regulatory mechanisms were evaluated using mRNA levels of TFs in colon cancer and normal colon tissue, which revealed 71 differentially expressed TFs (logFC of >1.0, FDR of <0.05) (Figures 3A,B). Nine of these TFs were associated with overall survival among colon cancer patients. Based on these findings, we created a regulatory network using the 9 TFs and 26 IRGs that were associated with survival (Figure 3C).

**TABLE 1:** General characteristics of survival-associated IRGs in patients with colon cancer.

Gene symbol	HR	HR.95L	HR.95H	*P*-value
PLCG2	1.78771957	1.329491	2.403883	0.000121
CHGA	1.01049537	1.005125	1.015895	0.000123
OXTR	1.40633083	1.181286	1.674249	0.000127
CD19	1.30216573	1.134585	1.494498	0.000172
CCL19	1.0235064	1.011097	1.036068	0.000189
CD79B	1.12218511	1.05124	1.197919	0.000541
TNFRSF13C	1.46244744	1.178929	1.814149	0.000546
CD22	1.21130031	1.086407	1.350552	0.000555
XCL1	2.15293999	1.381475	3.35522	0.000705
VIP	1.05870861	1.023354	1.095285	0.000994
FGF2	1.47456065	1.166011	1.864759	0.001186
TRAV8-2	3.32850327	1.583458	6.996669	0.001511
CR2	1.06879614	1.025544	1.113872	0.001596
IGHG1	1.00060367	1.000221	1.000986	0.00198
INHBE	3.61792417	1.593117	8.216207	0.002121
FGF9	2.42824902	1.358618	4.339995	0.00275
PTH1R	1.53935823	1.142105	2.074787	0.00462
IGHV2-70	1.01935188	1.005701	1.033188	0.005331
CCR7	1.17300899	1.046284	1.315083	0.006226
CD1B	0.04002157	0.003874	0.41346	0.006908
IL16	1.62583077	1.140763	2.317156	0.007178
SCG2	1.11004978	1.027623	1.199089	0.007999
SEMA3D	2.07808955	1.204535	3.585164	0.008569
PTGDS	1.03559807	1.008817	1.06309	0.00888
NGF	2.86414111	1.290971	6.354366	0.00965
CD40LG	1.7639811	1.14685	2.713197	0.009775

*HR, hazard ratio.*

### Construction and Validation of the Prognostic Risk Score

After excluding patients without survival data or with <30 days of data, we separated the remaining patients into a training dataset (*n* = 251, 70%) and a testing dataset (*n* = 104, 30%). The risk score was developed using the training dataset and validated using the testing dataset.

**FIGURE 4:**
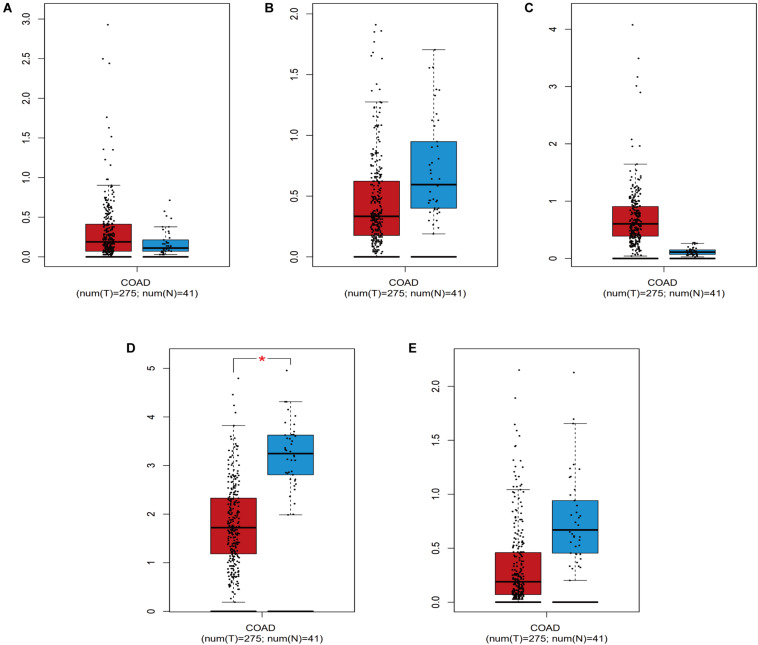
The expression level of the five IRGs included in the risk score in colon cancer patients compared to healthy people. **(A)** *CD1B*; **(B)** *NGF*; **(C)** *OXTR*; **(D)** *PLCG2*; **(E)** *XCL1*. COAD represent colon cancer. * represents statistical difference.

Screening of the survival-associated IRGs identified five relevant IRGs (*CD1B, XCL1, PLCG2, NGF*, and *OXTR*) via a Cox proportional hazards model, which were used to develop the risk score. The *CD1B* gene was considered a protective gene (coefficient of −3.62) and the other four IRGs were considered risk genes. The risk score for each patient was calculated as: risk score = (−3.62 × expression of *CD1B*) + (0.492 × expression of *XCL1*) + (0.52 × expression of *PLCG2*) + (0.876 × expression of *NGF*) + (0.203 × expression of *OXTR*). The expression level of each IRG was evaluated using the samples from the TCGA (Figure 4).

**FIGURE 5:**
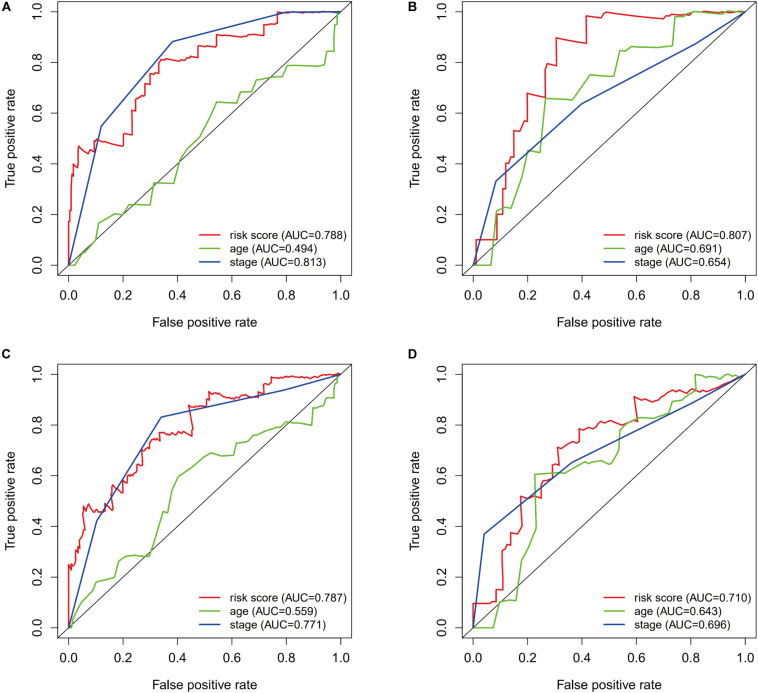
The prognostic value of the immunogenomic risk score. Survival-dependent receiver operating characteristic (ROC) curves for predicting 1-year survival **(A)** and 3-year survival **(C)** in the training dataset. The ROC curves for predicting 1-year survival **(B)** and 3-year survival **(D)** in the testing dataset.

The prognostic value of the risk score was evaluated using ROC curves for the training dataset. The AUC values were 0.788 for predicting 1-year survival and 0.787 for predicting 3-year survival (Figures 5A,C). The risk score appeared to provide excellent prognostic value relative to the AUC values for predicting survival based on tumor stage (1-year survival: 0.813, 3-year survival: 0.771). Validation using the testing dataset revealed AUC values of 0.807 for predicting 1-year survival and 0.710 for predicting 3-year survival (Figures 5B,D).

**FIGURE 6:**
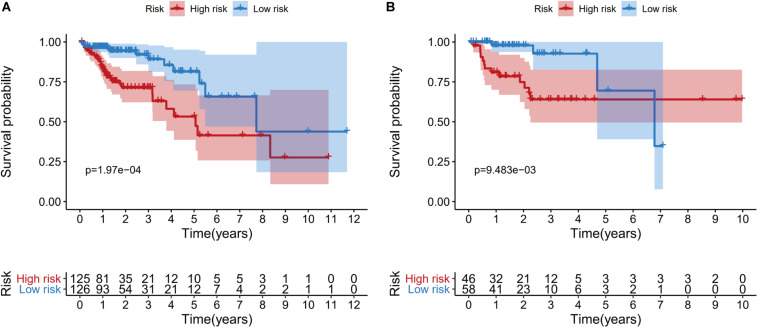
Kaplan-Meier curves of overall survival for the high-risk and low-risk patients in the training dataset **(A)** and the testing dataset **(B)**.

Patients from the training set were assigned to a high-risk group (*n* = 125) and a low-risk group (*n* = 126) based on the median risk score. The Kaplan-Meier survival curves revealed significantly better survival in the low-risk group than in the high-risk group (*P* = 1.97e−04) (Figure 6A). Patients from the testing set were also divided according to risk score (high-risk: 46 patients vs. low-risk: 58 patients), and the survival analyses also revealed significantly better survival in the low-risk group (*P* = 9.483e−03) (Figure 6B).

**FIGURE 7:**
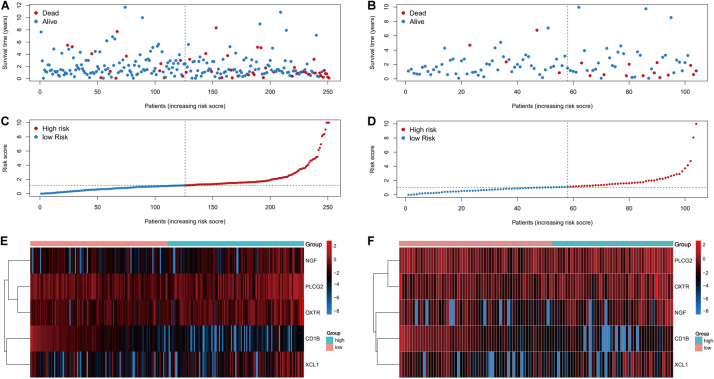
Prognostic analyses of high-risk and low-risk patients. **(A)** The risk score distribution, **(C)** survival status, and **(E)** risk gene expression in the training set. **(B)** The risk score distribution, **(D)** survival status, and **(F)** risk gene expression in the training set.

The risk score distributions, survival statuses, and risk gene expressions in the training and testing datasets are shown in Figures 7A–F. The high-risk group had clearly higher values for the risk score and the mortality rate. In contrast, the low-risk group had significantly higher expression of the protective gene (*CD1B*) and lower expression of the four risk genes. These results indicated that our risk model was capable of accurately predicting the prognosis of colon cancer patients. Multivariate Cox regression analyses also revealed that the risk score was an independent predictor of survival in the training and testing datasets, after adjusting for age, sex, and tumor stage (Table 2).

**TABLE 2:** Univariate and multivariate Cox regression analysis of clinicopathologic factors and risk score for OS in training and testing sets.

	Variable	Univariate		Multivariate	
			
		HR	*P*-value	HR	*P*-value
Training	Risk score (low risk vs. high risk)	1.020	3.82E−05	1.019	<0.001
	Age (≤65 vs. >65)	1.009	0.503	1.030	0.036
	Gender (male vs. female)	0.706	0.264	0.828	0.561
	Stage (I and II vs. III and IV)	2.628	6.46E−08	2.768	5.49E−08
Testing	Risk score (low risk vs. high risk)	1.348	0.001	1.390	0.005
	Age (≤65 vs. >65)	1.041	0.102	1.063	0.033
	Gender (male vs. female)	0.798	0.676	0.629	0.445
	Stage (I and II vs. III and IV)	2.620	0.003	2.380	0.009

*HR, hazard ratio; OS, overall survival.*

### Clinical Utility of the Prognostic Risk Score

**FIGURE 8:**
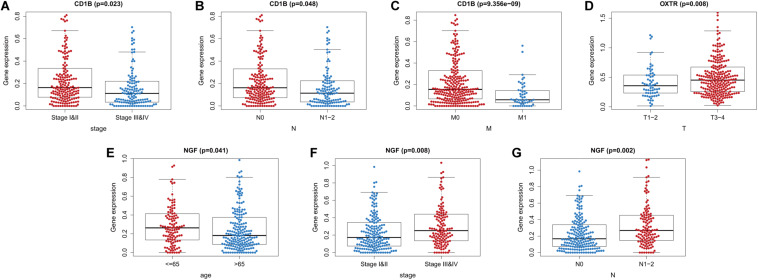
Relationships between the immune-related genes (IRGs) included in the risk score and clinical factors in colon cancer. **(A)** *CD1B* expression and tumor stage cases. **(B)** *CD1B* expression and lymph node metastasis. **(C)** *CD1B* expression and distant metastasis. **(D)** *OXTR* expression and T stage. **(E)** *NGF* expression and age. **(F)** *NGF* expression and tumor stage. **(G)** *NGF* expression and lymph node metastasis cases.

We also evaluated whether the risk score could predict progression of colon cancer by evaluating the IRGs’ relationships with clinical variables (age, sex, and tumor stage) (Table 3). The expression of *CD1B* was significantly lower in cases involving advanced-stage disease, lymph node metastasis, and distant metastasis (Figures 8A–C). The expression of *OXTR* was notably higher in cases with an advanced T classification (Figure 8D). The expression of *NGF* was markedly higher in cases involving elderly patients, advanced-stage disease, and lymph node metastasis (Figures 8E–G).

**TABLE 3:** Relationships between risk factors (the risk score and the risk genes) and clinical variables in colon cancer.

Gene symbol	Age (≤65/>65)	Sex (male/female)	Tumor stage (I and II/III and IV)	T stage (T1-2/T3-4)	M stage (M0/M1)	N stage (N0/N1)
CD1B	−1.051 (0.294)	1.49 (0.137)	2.277 (0.023)	1.63 (0.106)	5.928 (9.36e−09)	1.987 (0.048)
XCL1	0.518 (0.605)	1.238 (0.217)	−1.122 (0.263)	−0.338 (0.736)	−1.261 (0.213)	−1.02 (0.309)
PLCG2	0.209 (0.835)	0.368 (0.713)	−1.423 (0.156)	−0.871 (0.385)	−0.871 (0.387)	−1.822 (0.070)
NGF	2.063 (0.041)	0.868 (0.386)	−2.697 (0.008)	−1.911 (0.059)	−1.84 (0.071)	−3.061 (0.002)
OXTR	0.128 (0.898)	−1.203 (0.230)	−1.529 (0.128)	−2.654 (0.008)	−0.918 (0.363)	−1.522 (0.130)
Risk Score	1.062 (0.291)	−0.975 (0.331)	−1.453 (0.149)	−1.624 (0.106)	−1.323 (0.192)	−1.451 (0.149)

**FIGURE 9:**
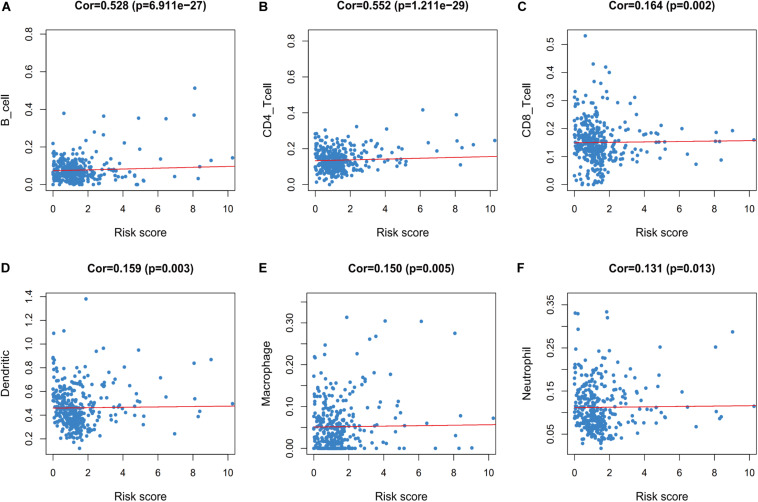
Relationships between the immune-related risk score and the infiltration abundances of six types of immune cells. The correlation was performed by using Pearson correlation analysis. **(A)** B cells; **(B)** CD4 T cells; **(C)** CD8 T cells; **(D)** dendritic cells; **(E)** macrophages; and **(F)** neutrophils.

Furthermore, we evaluated whether the risk score could reflect the tumor microenvironment, based on the correlation between the risk score and immune cell infiltration in the colon cancer patients. Higher risk scores were correlated with increasing values for tumor-infiltrating immune cells, which included B-cells, CD4^+^ T-cells, CD8^+^ T-cells, neutrophils, macrophages, and dendritic cells (all *P* < 0.05) (Figure 9).

## Discussion

This study revealed that 5 IRGs were associated with survival among patients with colon cancer. To the best of our knowledge, this is the first study to construct a reliable immunogenomic model for predicting overall survival among patients with colon cancer.

Immune evasion is an indispensable step in cancer development and this process is mediated by various IRGs (Concha-Benavente and Ferris, 2017), which prompted us to consider the role of IRGs in prognostication. We screened differentially expressed IRGs in colon cancer and identified 173 upregulated IRGs and 311 downregulated IRGs. These results indicated that IRGs were closely related to the development of colon cancer, which agrees with previously reported data. As expected, the Gene Ontology analyses revealed that the most common biological processes for the IRGs involved “immune response”, while the Kyoto Encyclopedia of Genes and Genomes analyses revealed that the differentially expressed IRGs were related to cytokine-cytokine receptor interactions. In this context, cytokines are mainly expressed by immune cells and tumor cells, and can alter anti-tumor immunity and tumor progression (Zhang et al., 2018; Weinstein et al., 2019). Some cytokines have also been used for cancer therapy, such as IL-8 (Ning and Lenz, 2012) and IL-6 (Wang et al., 2019). Thus, our findings may provide a basis for future studies regarding ways to target these IRGs.

Previous studies have indicated that TF dysregulation, which significantly modifies gene expressions, was related to the progression of colon cancer (Laissue, 2019). For example, the FOXE1 TF is a critical tumor inhibitor that regulates tumor growth and glycolysis by suppressing HK2 in CRC (Dai et al., 2020). The ZBP-89 TF can also drive a feed-forward loop involving β-catenin expression in CRC (Essien et al., 2016). Thus, to explore the regulatory mechanisms for the IRGs, we identified differentially expressed TFs in colon cancer and established a network involving the differentially expressed TFs and IRGs. We identified nine important TFs, with CBX7, FOXP3, LMO2, MAF, MYH11, NR3C1, and SPIB dominating the network. These results suggested that TFs could influence the effects of IRGs on survival outcomes.

Among the differentially expressed IRGs, we identified 5 IRGs (*CD1B, XCL1, PLCG2, NGF*, and *OXTR*) for inclusion in the risk score, and previous studies have indicated that these genes were related to immune processes and cancer progression. The stability of the risk score was evaluated based on the expression level of each IRG, which were stably expressed in the colon specimens. The *CD1B* gene encodes a member of the Group 1 CD1 family of transmembrane glycoproteins, which present a wide array of self and foreign lipid antigens to T-cell receptors (Shahine, 2018). Previous reports have indicated that *CD1B*-restricted self-lipid-reactive T-cells responded more potently to lipid from tumor cells than to an equivalent amount of lipids from normal cells, and the adoptive transfer of these T-cells into mice resulted in tumor control (Bagchi et al., 2016). Furthermore, *CD1B* expression was related to the prognosis for localized prostate cancer (Lee et al., 2019), and *CD1B* was detectable within mononuclear cells from liver tumor specimens, but was not expressed in healthy livers (Kenna et al., 2007).

The *XCL1* gene encodes a C class chemokine that is alo known as lymphotactin (Lei and Takahama, 2012). The ability of *XCL1* to contribute to the anti-tumor activity via attracting DC1s has been demonstrated in a mouse tumor model (Böttcher et al., 2018). In addition, *XCL1* expression is related to the number of tumor-infiltrating CD8^+^ T-cells and PD-L1 expression on ovarian tumor cells, which indicated that *XCL1* might be a biomarker for anti-PD1/PD-L1 immunotherapy (Tamura et al., 2020).

The *PLCG1* gene encodes an intracellular signaling molecule that is positioned at the convergence of various signaling pathways for cell proliferation, migration, and invasion (Wells and Grandis, 2003). Dysfunction of *PLCG1* is closely associated with inflammation, immune disorders, and cancer (Koss et al., 2014), and *in vitro* testing revealed that *PLCG1* mediated high glucose levels and insulin-induced cell proliferation and migration in SW480 colon cancer cells (Tomas et al., 2012). The expression of *PLCG1* also promoted hepatoma cell carcinogenesis *in vitro* and *in vivo* (Tang W. et al., 2019).

The *NGF* gene encodes a growth factor that can be released into or produced by the tumor microenvironment (Bradshaw et al., 2015). Expression of *NGF* influences the activities of various immune cells, including macrophages, granulocytes, T-cells, B-cells, NK cells, and eosinophils. These cells can also synthesize, store, and release consistent amounts of NGF, which suggests that NGF may influence the anti-tumor immune response (Aloe et al., 2016). Moreover, NGF activates breast cancer stem cells through the promotion of epithelial-mesenchymal transition and by increasing the number of symmetric divisions, which indicates that NGF is involved in the self-renewal and plasticity of cancer stem cells (Tomellini et al., 2015). Expression of *NGF* also increases angiogenesis via the COX-2/PGE2 signaling axis in epithelial ovarian cancer (Garrido et al., 2019).

The *OXTR* gene encodes the oxytocin receptor, and the oxytocin/*OXTR* axis is known to decrease the sensitivity of macrophages to lipopolysaccharides, with lower expression of inflammatory cytokines (e.g., IL-1β, IL-6, and TNF-α) and to increase the sensitivity to IL-4 stimulation in the intestinal microenvironment (Tang Y. et al., 2019). Thus, the oxytocin/*OXTR* axis may be useful for inhibiting CRC development via down-regulating immunosuppressive proteins (FAPα and CCL-2) (Ma et al., 2019).

The risk score provided AUC values of 0.788 for predicting 1-year survival and 0.787 for predicting 3-year survival, which indicated the score had good prognostic ability. We also divided the patients according to the median risk score, and observed remarkable differences in their survival curves, which confirmed that the risk score was useful for identifying patients with a high risk of death. Nevertheless, medical improvements have allowed even some advanced cancer patients to achieve long-term survival (Li et al., 2019), and frequent follow-up with active management may still be useful for patients with stage IV colon cancer (Karoui et al., 2011; Becerra et al., 2015). In clinical practice, tumor staging can be used to guide patient classification and more personalized treatment (Lu et al., 2015; Babaei et al., 2018), although patients with the same disease stage can still experience different outcomes (Mayanagi et al., 2018). Thus, for patients with the same colon cancer stage, our risk score may be useful as an auxiliary tool to identify patients with a high risk of death.

We evaluated the relationship between the risk score and tumor-infiltrating immune cells, which revealed a positive correlation with the number of infiltrating immune cells. In this context, tumor immune evasion is mediated by B-cells, CD4^+^ T-cells, CD8^+^ T-cells, neutrophils, macrophages, and dendritic cells (Liu and Cao, 2016; Spranger, 2016), which are closely related to tumorigenesis, progression, and metastasis (Berntsson et al., 2016; Liu and Cao, 2016; Väyrynen et al., 2016; Prizment et al., 2017; Van den Eynde et al., 2018). Thus, our risk score might be useful for evaluating the patient’s immune status and guiding treatment.

Previous immunogenomic models have shown favorable predictive value in this setting. Wu et al. developed a prognostic model using 19 IRG pairs for colon cancer (Wu et al., 2019), while Ge et al. developed prognostic models for stage I–II colon cancer (using 2 IRGs) and stage III–IV colon cancer (using 3 IRGs) (Ge et al., 2019). However, the IRGs identified in those studies were different from the IRGs in our risk score, which may be related to differences in the studies designs and patient populations. Moreover, we performed comprehensive analyses to identify the IRGs for the model, based on multiple algorithms, univariate Cox analyses, lasso regression analysis, and a Cox proportional hazards model.

Our study has several limitations. First, the risk score could not be validated based on clinical practice because of a lack of clinical specimens, and our risk score needs to be validated in other well-powered studies. Second, the predictive value of the risk scores was not discussed in the left-sided or right-sided colon cancer, respectively. We will collect colon cancer patients with tumor sites to verify our results in future study. Furthermore, the effects of the IRGs on colon cancer development remain unclear, although cancer progression has been linked to *CD1B* (Lee et al., 2019), *XCL1* (Chou et al., 2020; Tamura et al., 2020), *PLCG1* (Wells and Grandis, 2003), *NGF* (Retamales-Ortega et al., 2017; Garrido et al., 2019), and *OXTR* (Ma et al., 2019). Therefore, the underlying mechanisms require further investigation using *in vivo* and *in vitro* experiments.

## Conclusion

The present study developed an immunogenomic risk score that appears to have promising value for prognostication in cases of colon cancer. While it will not replace traditional cancer staging, it may help refine prognostication among patients who had the same disease stage. However, further prospective studies are needed to evaluate the score’s prognostic accuracy and clinical utility in the management of colon cancer.

## Data Availability Statement

The datasets generated for this study can be found in the datasets (Transcriptome expression profiles and Clinical data for the corresponding patients), for this study can be found in the [The Cancer Genome Atlas (TCGA) data portal] (https://cancergenome.nih.gov/), the datasets (IMMUNE-RELATED GENE DATA) for this study can be found in the [Immunology Database and Analysis Portal (ImmPort) database] (https://immport.niaid.nih.gov), the datasets (Immune infiltrate data) for this study can be found in the (Tumor Immune Estimation Resource) (https://cistrome.shinyapps.io/timer/).

## Author Contributions

HZ and CQ: study conception, design, and performance, as well as writing of the report. XG: provided critical suggestions regarding the figures and tables. LZ: data collection. HG: led the research team. All authors read and approved the final manuscript.

## Conflict of Interest

The authors declare that the research was conducted in the absence of any commercial or financial relationships that could be construed as a potential conflict of interest.

## Footnotes

1 https://cancergenome.nih.gov/

2 https://immport.niaid.nih.gov

3 http://cistrome.org/

4 https://cistrome.shinyapps.io/timer/

5 http://david.ncifcrf.gov/home.jsp

6 http://kobas.cbi.pku.edu.cn/kobas3

7 http://gepia.cancer-pku.cn/

8 http://www.Rproject.org
